# Effects of Acute and Chronic Physical Exercise on Esports Performance: A Systematic Review

**DOI:** 10.1186/s40798-026-01034-9

**Published:** 2026-05-23

**Authors:** Felix Wachholz, Fynn Coenen, Martin Zhang, Heiko Heidenreich

**Affiliations:** 1 Department of Sport Science, University of Innsbruck, Fürstenweg 185, 6020 Innsbruck, Austria; 2 Research and Strategy Services Division, LMU Munich, Elisabethplatz 22, 80796, Munich, Germany

**Keywords:** Physical activity, Training, Professional gaming, Competitive, Improvement, Intensity, Duration

## Abstract

**Background:**

Recent evidence suggests that structured physical exercise (PE) may enhance electronic sports (esports) performance by targeting physiological and cognitive mechanisms similar to those known from traditional athletic training. Esports require rapid decision-making, sustained attention, and fine motor abilities—capacities strongly influenced by regular PE. However, research on the effectiveness and practical implementation of acute and chronic PE interventions on esports performance remains fragmented.

**Methods:**

In accordance with the Preferred Reporting Items for Systematic Reviews and Meta-Analyses (PRISMA) guidelines, searches were conducted across three major databases, supplemented by snowballing and hand-searching. Studies meeting the Population, Intervention, Comparison, Outcome, and Study Design (PICOS) criteria were subsequently included.

**Results:**

The review synthesized findings from twelve experimental studies investigating acute and chronic PE interventions with e-athletes of different expertises. The interventions included mostly high-intensity interval-training using cycling, walking, treadmill protocols, and exergaming using virtual reality applications, such as Beat Saber. Eight studies analyzed acute effects of PE interventions and typically ranged from 6 to 30 min, while five studies evaluated chronic PE programs and lasted 8–10 weeks with sessions of about 30 min three times per week (one study analyzed both). Across studies, both acute and chronic PE interventions produced beneficial effects on esports-related outcomes. Acute PE consistently improved performance metrics, e.g., executive function, reaction time, aiming accuracy, and elimination rate. Chronic PE interventions demonstrated mixed results: several studies reported gains in executive functions, overall coordination, and grip strength, whereas others found no significant effects on direct esports-related performance measures. Importantly, rest intervals of up to 30 min between exercise and play did not diminish positive effects, underlining the practical feasibility of PE as part of tournament preparation routines.

**Conclusions:**

Current evidence supports the systematic inclusion of structured PE in esports training. PE might not only promotes health and stress resilience but also improves cognitive and perceptual abilities directly linked to competitive esports and gaming performance. Future research should standardize esports performance measures, investigate the specific mechanisms of transfer between physical and virtual training, and validate long-term interventions to establish evidence-based PE protocols for e-athletes.

**Registration:**

The systematic review was prospectively registered through PROSPERO (CRD420251028699).

## Key points

- Acute and chronic physical exercise (PE) interventions have been shown to enhance esports performance, particularly in cognitive domains, such as executive function, attention, reaction time, visuomotor control, but also game performance.
- Acute PE appears to consistently improve performance, while chronic PE interventions demonstrate more heterogeneous effects, improving physical and cognitive capacities but not always direct esports-related performance parameters.
- Exercise intensity, duration, and type appear less critical than regular engagement and skill specific task similarity.
- The beneficial effects of PE persist even after rest intervals of up to 30 minutes, highlighting its practical applicability as a pre-competition preparation tool for e-athletes.

## Introduction

Electronic sports (esports) have emerged as a global phenomenon, attracting millions of viewers worldwide and establishing itself as one of the fastest-growing forms of entertainment [1]. Particularly among younger generations, esports and gaming serves not only as a popular leisure time activity [2], but also as a medium of identity formation and community engagement [3]. Drawing on earlier definitions by Jenny et al. [4], esports refers to “organized video game competitions, also commonly referred to as cybersport, virtual sport, and competitive gaming” [5, p. 1], in which individuals or teams compete using motor, strategic, or cognitive skills, often for rewards and in front of an audience or via broadcasting [6]. This competitive and performative dimension distinguishes esports from recreational gaming. Professional players—commonly referred to as “e-athletes” or “e’athletes” [7]—can nowadays generate substantial earnings, where significant financial stakes are involved, professionalism in terms of structured leagues [8], associations [9], and athlete development typically follows [10, 11].

Due to the rather fine motor activities, the classification of esports as “sport” has been the subject of considerable debate [12, 13], one that has gained additional momentum in light of the announcement of the inaugural Olympic Esports Games scheduled for 2027 [14], although details regarding their timing and format remain under development [15]. Critics argue that esports lacks direct physicality and whole-body control, does not adequately foster holistic human development, suffers from fragmented institutional structures, and resembles sport more superficially than essentially [16]. Proponents, by contrast, emphasize that esports involves uniquely human skills, including fine motor control, cognitive performance, and strategic thinking. They also argue that physicality should not be narrowly defined by gross motor movements, as digital embodiment and dexterity constitute meaningful forms of physical engagement [17]. Furthermore, despite its relatively young age, esports already exhibits many of the hallmarks of institutionalization, such as leagues, governance structures, and codified rules, even if these remain less consolidated than in traditional sports [8]. Moreover, Naraine et al. (2021) propose that the very concept of sport must evolve alongside cultural and technological transformations, rather than remain bound to an exclusively Olympic model from the past. Especially, as the traditional model centered on physical movement and measurable bodily performance [17].

Beyond its developing professionalism, economic and cultural significance, esports has also been discussed in terms of its potential cognitive benefits for e-athletes, which may provide a basis for leveraging physical exercise (PE) as a means of performance enhancement. Research on video gaming has demonstrated positive effects on cognitive functioning, such as attentional control, working memory, and problem-solving skills [18–20]. Importantly, these effects may vary depending on the specific game genre [21], e.g., action [22] vs. strategy games [23], with action and first-person shooter (FPS) games being more strongly associated with improvements in attentional control and visuospatial processing [24], whereas strategy-based genres such as real-time strategy (RTS) and multiplayer online battle arena (MOBA) games are more closely linked to enhancements in working memory, planning, and cognitive flexibility [23].

As esports performance is inherently linked to a range of cognitive abilities, e.g., fast decision-making, divided attention, and complex problem-solving under pressure, and which are critical for processing rapidly changing in-game information and executing precise motor responses, these benefits are likely transferable to esports [25, 26]. PE is suggested to improve cognitive functioning as well as esports-related abilities [27, 28] and a growing body of literature supports the notion that PE positively influences cognitive abilities and performance [27], with respect to the type, duration, and intensity of exercise playing important roles [28]. PE has been associated with acute and chronic enhancements in cognitive functioning, particularly in domains, such as executive functions, attention, and processing speed [29, 30]. Acute bouts of exercise have been shown to transiently improve attentional resources and reaction speed [31], which might be important for FPS e-athletes [24]. On the other hand, long-term exercise interventions are linked to structural and functional adaptations supporting executive control and cognitive flexibility [29], particularly important in RTS-games [23]. These cognitive benefits suggest a potential mechanism through which PE may positively influence esports performance, particularly in cognitively demanding game environments. Distinguishing between acute and chronic effects of PE represents an important consideration when developing optimized training strategies for e-athletes, just as exercise density and periodization are critical for e-athletes preparation [32].

Recent research has increasingly examined the role of physical activity and physical fitness within esports, highlighting both health-related and performance-relevant implications. Empirical studies indicate that esports players often display heterogeneous and, in some cases, insufficient levels of physical activity, alongside prolonged sedentary behavior, which may negatively affect health and potentially performance [33, 34]. Very recent evidence further highlights the growing interest in the relationship between physical fitness and esports performance. A study published after the final search date of the present review investigated associations between PE characteristics and esports-related outcomes, providing additional support for the notion that physical conditioning may play a role in cognitive and/or in-game performance for FPS e-athletes. Although not included in the current systematic synthesis due to the predefined search period, these findings align with emerging intervention-based and observational evidence suggesting that targeted PE could complement traditional esports practice [35]. Applied research and case-based evidence indicate that esports organizations are increasingly integrating PE into training routines to enhance performance readiness, prevent injury, and support long-term player development [36]. More recent work further highlights the expanding interdisciplinary support systems in esports, including physical conditioning as part of holistic performance programs [37]. Nevertheless, the role of PE within esports remains insufficiently understood, and e-athletes often over-report their physical activity levels [38], despite being aware of the potential negative health effects associated with esports [39]. While the physical component in esports is mainly restricted to fine motor abilities, such as mouse and keyboard use, console controllers, or touchscreen interaction, emerging discussions highlight that e-athletes may still experience high physiological strain, for instance, through elevated stress, prolonged sitting, and repetitive strain injuries [40]. However, PE as peformance improving factor may play a crucial role to potentially benefit e-athletes in two important ways: (i) by directly enhancing performance capacity and (ii) by reducing the risk of overuse injuries and sedentary lifestyle-related health problems [36]. If empirical evidence demonstrates that structured PE can significantly improve esports performance, PE could become an essential component of e-athletes’ training schedules. Moreover, professional e-athletes engaging in regular PE could have a demonstration effect [41], serving as role models and encouraging healthier behaviors among recreational players [42] and especially youth audiences [43].

Given the rapidly evolving and still fragmented nature of research on PE in esports, a systematic review approach is warranted. Existing studies vary substantially in terms of design, populations, intervention characteristics, and outcome measures, which limits the ability to draw coherent conclusions from individual investigations. Moreover, evidence on acute and chronic effects of PE on esports performance and player health remains dispersed across disciplines and methodological approaches. A systematic review enables a structured and transparent synthesis of this heterogeneous body of literature, allowing for the identification of consistent patterns, methodological gaps, and directions for future research. By integrating findings across studies, this approach provides a more comprehensive understanding of the role of PE in esports than a single empirical study could achieve and supports the development of evidence-based recommendations for both research and practice.

Hence, the aim of the current article is to synthesize available evidence regarding the effects of both acute and chronic PE on the performance of e-athletes. By summarizing the current state of knowledge, this review seeks to provide guidelines and practical implications for e-athletes and practitioners on how to strategically incorporate PE to optimize performance and safeguard long-term health.

## Methods

The present work was conducted as a systematic literature review following the PRISMA 2020 statement (Preferred Reporting Items for Systematic Reviews and Meta-Analyses [44, 45]). The aim of this review was to systematically examine the impact of acute and chronic PE inteventions on the performance of e-athletes, with a particular focus on objective esports performance parameters. The review was registered in the Prospero [46] database (CRD420251028699). Deviations from the preregistered protocol included a refinement of the search strategy through the addition of hand-searching, as well as adjusted inclusion criteria to better account for the heterogeneous esports performance parameters assessed across studies. Hence, the literature search was based on three complementary strategies: (i) a traditional systematic database search using the databases Web of Science, PubMed and EBSCOhost and (ii) snowballing (both forward and backward), whereas in forward snowballing, citation tracking was applied, while in backward snowballing, the reference lists of included studies were analyzed. In addition (iii) a targeted hand-search of specialized esports journals that are not indexed in the mentioned databases (e.g., International Journal of Esports, Journal of Electronic Gaming and Esports, Annals of Esports Research) was also conducted. These procedures were implemented to minimize the risk of publication bias and ensure that relevant domain-specific studies not captured by traditional databases were considered. The PRISMA 2020 statement explicitly recommends the use of complementary search strategies such as hand-searching of key journals or reference lists to enhance the comprehensiveness of evidence retrieval [44]. Following this guidance, our approach aimed to achieve a more exhaustive coverage of the interdisciplinary esports performance literature. Identified studies were screened and selected according to predefined inclusion and exclusion criteria and were subsequently analyzed in detail. Data collection, processing, and analysis were performed using Excel (Microsoft Excel 2019). To evaluate the methodological quality of the included studies, the established checklist by Downs and Black (1998) [47] was applied.

### Study Inclusion and Exclusion Criteria

A Population, Intervention, Comparator, Outcome, and Study Design (PICOS) framework was developed and applied to determine the suitability of studies, with those not meeting the following criteria being excluded [48]: population: male or female e-athletes engaged in regular competitive play; intervention: acute or chronic PE interventions requiring participants to engage in full-body movement or exercise; comparator: pre-to-post changes in outcome measures induced by an acute PE session and/or a chronic PE intervention period; outcomes: esports performance indicators (e.g., kill/death ratio, targets eliminated, accuracy, win/loss rate, task completion time) and/or esports-related cognitive (e.g., executive functions, reaction time, anticipation) and motor (e.g., coordination, grip strength) abilities; study design: studies that were published in English in peer-reviewed journals, applied empirical methods, and followed a randomized controlled trial (RCT) or comparable experimental design. Furthermore, eligible studies had to examine esports performance as the dependent variable and include a clearly described PE intervention. No temporal restrictions were applied in the search strategy, and studies were considered for inclusion regardless of their publication date.

**Fig. 1:**
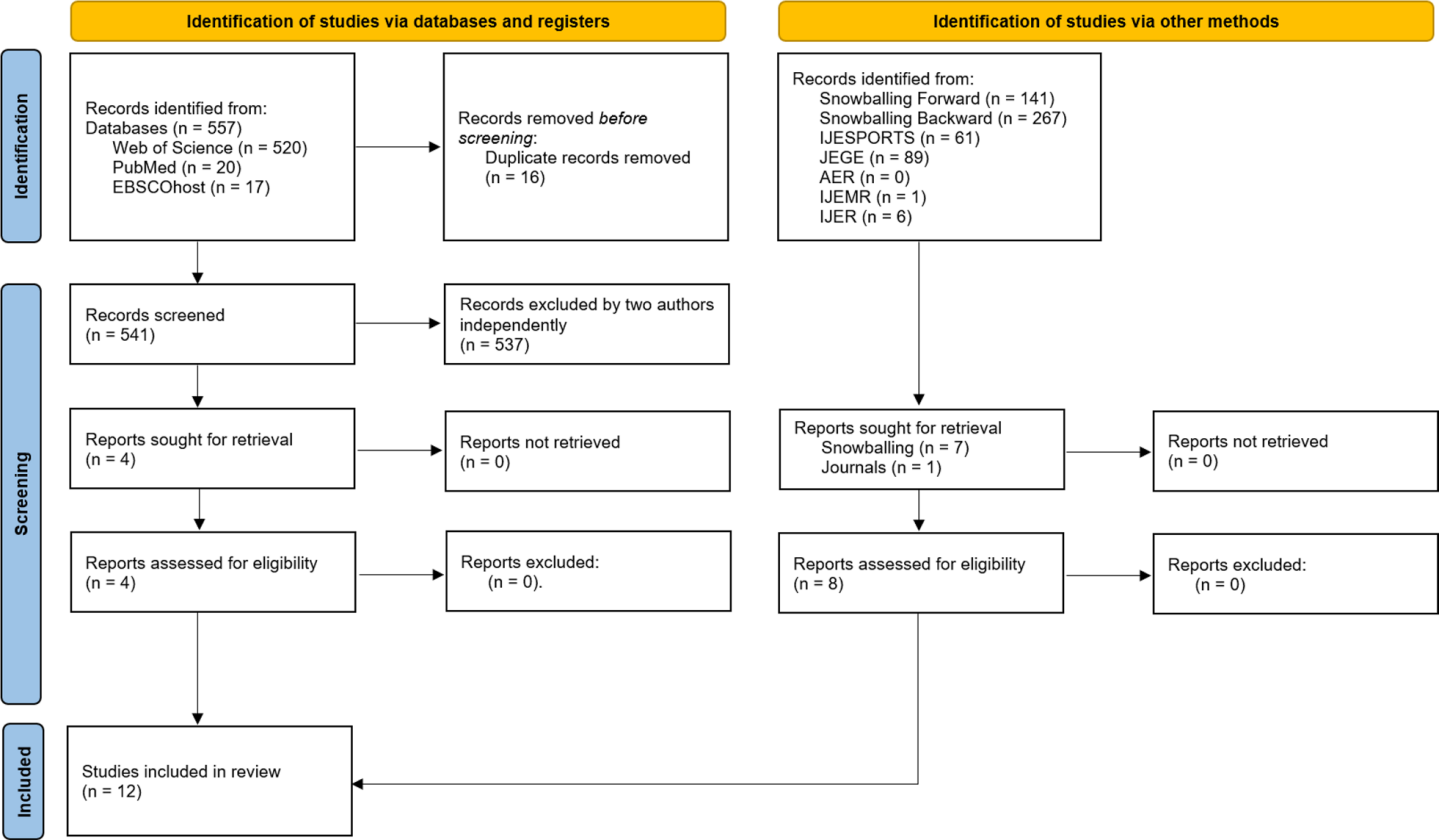
PRISMA flowchart. *IJESPORTS* International Journal of Esports, *JEGE* Journal of Electronic Gaming and Esports, *AER* Annals of Esports Research, *IJEMR* International Journal of eSports Multidisciplinary Research, *IJER* International Journal of eSports Research

Studies were excluded if they were purely theoretical in nature, lacked a direct connection to PE or esports, or did not employ empirical methods with quantified esports performance parameters. Ultimately, all studies meeting the inclusion criteria were incorporated into the final selection. In addition to randomized controlled trials, quasi-experimental and pre–post pilot studies were included when they implemented a clearly defined PE intervention involving e-athletes or players under competitive gaming conditions. This decision was made to capture the emerging body of experimental evidence in the field, which often comprises pilot or exploratory studies with limited sample sizes or non-randomized allocation but still provides valuable mechanistic or performance-related data. To document the selection process, a PRISMA flowchart (Fig. 1) was created.

### Search Strategy for Identification of Studies

The systematic literature search was conducted between the 1st and 15th October 2025 across the scientific databases Web of Science, PubMed, and EBSCOhost. The databases were searched to cover the interdisciplinary field of esports performance: Web of Science for multidisciplinary sport science, psychology, and computer science journals; PubMed for biomedical and cognitive performance studies; and EBSCOhost for applied sport science and psychology literature, including coaching, training, and performance research. The search string was iteratively adapted and optimized to account for the wide range of spellings and terminologies within the field of esports. Due to the novelty of esports as a research area, no standardized terminology has yet been established in the scientific literature. Based on the article by Bubna et al. [7] the final search string was defined as follows:

- *(esport OR e-sport OR esports OR E-Sports OR cybersport OR cyber-sport OR competitive gaming OR professional gaming OR videogaming OR video-gaming OR elite OR competition OR competitive OR competitors OR professional) AND (eathlete OR e-athlete OR e’athlete OR eathletes OR e-athletes OR e’athletes OR esportplayers OR esport-player OR esportsplayer OR esports-player) AND (performance) AND (physical OR exercise OR endurance OR strength OR psychological OR mental) AND (intervention OR training program OR Randomized controlled trial OR Randomized controlled trial)*.

After the removal of duplicates, potentially relevant studies were screened against predefined inclusion criteria through a two-step process: (i) title and abstract screening followed by (ii) full-text screening. All studies meeting the criteria were subsequently included and subjected to detailed content analysis. In addition, following PRISMA guidelines [45], forward and backward snowballing was applied, starting with the highly cited seed article by Zhang et al. [49] and extended to all included studies, ensured maximal coverage. As a third step a hand-search [45] in the esports-related journals International Journal of Esports (IJESPORTS), Journal of Electronic Gaming and Esports (JEGE), Annals of Esports Research (AER), International Journal of eSports Multidisciplinary Research (IJEMR) and International Journal of eSports Research (IJER) was applied, by screening all published articles by the 15th of October 2025.

### Quality Assessment and Analysis

The methodological quality of the included studies was assessed using the Downs and Black checklist [47]. This instrument allows for a differentiated evaluation of methodological quality in both randomized and non-randomized studies of health interventions. The checklist consists of 27 items categorized into four domains: reporting, external validity, internal validity, and statistical power. A maximum score of 32 points can be achieved, as item 5 allows for up to 2 points and item 27 for up to 5 points [47]. The checklist was systematically applied to the full texts of the selected studies. Each domain was assessed based on the available information, thereby considering both the strength of evidence and potential sources of bias, which are crucial for interpreting the findings. The quality ratings were documented in tabular form using Excel (Microsoft Excel 2019). To ensure reliability, three authors independently performed the assessment. Any discrepancies were discussed, and the mean score of all evaluations was used for the final rating. For the analysis, data extraction was performed for all included studies based on predefined categories and documented in an Excel (Microsoft Excel 2019) sheet. The following categories were recorded: author, title, year, study design, journal, method, participants, country, definition of esports, control group, type of intervention, performance parameters, measurement method, type of effect, results, additional findings, implications, and limitations. Particular attention was paid to identifying similarities and differences across studies, as well as documenting significant effects of PE on esports performance. Given the heterogeneous nature of the included studies, encompassing multiple study designs, a cross-design assessment tool was considered most appropriate to ensure comparability across studies. While design-specific instruments such as RoB 2 [50] and ROBINS-I [51] represent current best-practice approaches for risk-of-bias assessment, their combined use would have introduced additional complexity and potentially reduced comparability, particularly in the context of a relatively small and methodologically diverse evidence base. Therefore, the Downs and Black checklist was selected as the most suitable approach to provide a consistent and comprehensive assessment of methodological quality in this review.

## Results

In total, 557 studies were identified across three databases. Of these, 16 studies were duplicates and subsequently removed, resulting in 541 unique records. After title/abstract and full-text screening conducted by two authors independently, 4 studies met the inclusion criteria. The forward and backward snowballing lead to in total 141 citing articles and 267 references which screened against the same inclusion criteria, resulting in 7 additional eligible studies. The conducted hand-search resulted in 1 more additional article found in the journal International Journal of Esports (IJESPORTS). Ultimately, 12 studies fulfilled the inclusion criteria and were included in the final synthesis (see PRISMA flowchart, Fig. 1).

### Characteristics of the Included Studies

As shown in Table 1, the studies were published between 2020 and 2025, with most appearing in 2024. The mean Downs and Black score [47] was 22.3 ± 2.1, which is considered good to moderate article quality. The inter-rater reliability (ICC, two-way random model with absolute agreement) of the Downs and Black ratings was good (ICC[2,k] = 0.826, 95% CI [0.502–0.947, *p* < 0.001). The single-measure ICC indicated moderate agreement between raters (ICC[1, 2] = 0.613), while Cronbach’s *α* = 0.878 suggested high internal consistency among raters. Sample sizes ranged from *n* = 6 [52] to *n* = 93 [53]. Seven of the twelve studies focused on acute PE effects on esports performance [49, 54–59], while four studies addressed effects of chronic PE [52, 53, 60, 61], and one study evaluated both [62]. Training duration per session varied considerably, from 4:30 min [58] to 40 min [61], with intensities ranging from moderate to maximal effort. Four studies applied high-intensity interval training (HIIT) [52, 55, 59, 61], three implemented moderate endurance training [49, 56, 57], two used the VR (virtual reality) application Beat Saber [53, 60], one used walking [54] and one maximal cycling sprints [58] as intervention. Most studies used randomized controlled trial designs in various formats; two applied different designs being a within-subject pre–post trial without control [52] or using a quasi-experimental with age-matched controls [58]. Participant samples ranged from amateur e-athletes [53, 55, 57–62], to semi-professionell [54, 56], and professionell e-athletes [49, 52]. Although Mancı et al. (2024) and Nicholson et al. (2024) did not conduct randomized controlled trials, both studies were retained in the review as controlled or quasi-experimental evidence to complement randomized trials, while their methodological limitations (absence of randomization and/or control conditions) were accounted for in the quality and risk-of-bias assessment.

**Table 1:** Key characteristics of the included studies

Study	Participants	PE	Game/level	Measures	Intervention	Design	Major finding	D&B [mean]
De las Heras et al. [55]	*N* = 18 16 men 22.0 ± 3.0 y	A	LoL Amateurs	Game performance (targets eliminated, accuracy = single-attack eliminations) Positive and negative affect schedule (PANAS) VO₂_peak_	HIIT cycling 15 min 80–85% of maximal ergometer wattage (5 × 1-min intervals)	Randomized, counterbalanced within-subject crossover design	HIIT session followed by a 20-min rest led to a 9% improvement in target elimination ability (p = 0.027) and a 75% increase in attack accuracy (p = 0.019)	22.0
DiFrancisco et al. [54]	*N* = 21 12 men 20.8 ± 2.6 y	A	Multi-game* Semi-pros	Game performance (Kill/death ratio, win/loss rate) Tower of London, stroop test Borg RPE scale	During 2-h FPS gaming sessions 6-min walking break 6-min supine rest break “Light” intensity effort	Randomized repeated-measures crossover design	Walk break significantly improved executive function (p = 0.020) compared to rest and continuous play, while rest breaks led to the poorest performance. No differences were found in Stroop test results or gaming outcomes. About 70% of players perceived the walk break as beneficial, whereas rest breaks were often seen as tiring. No gender effects were observed	22.3
Dos Santos et al. [56]	*N* = 20 20 men 19.8 ± 2.5 y	A	CS:GO Semi-Pros	Game performance (time to eliminate 250 targets, accuracy = shots per kill) Heart rate Borg RPE scale	Moderate cycling and/or cognitive warm-up 22 min 60–70% of HRₘₐₓ	Randomized, counterbalanced within-subject crossover design	PE significantly reduced task completion time by 7.6% (p < 0.050) compared with all other conditions, while accuracy remained unchanged and precision was superior in comparison. Neither cognitive exercises alone nor in combination with PE improved player performance	22.3
Gaige et al. [57]	*N* = 20 20 men 24.0 ± 6.0	A	Rocket league Amateurs	Game performance (1v1 tournament outcomes, wins, goals, goal %, saves, score, shots) Stroop test and Trail Making Test (TMT-A, TMT-B) Visual analog scales for concentration, stress, fatigue, individual and opponent performance, match difficulty	Moderate cycling 20 min 60–70% of HRₘₐₓ	Randomized, counterbalanced crossover design	PE resulted in greater initial fatigue (p = 0.002), higher average heart rates throughout the tournament (p < 0.001), lower perceived individual performance in series 1 (p = 0.030), lower overall perceived opponent performance (p = .030), and an improved goal-scoring rate in series 3 (p = 0.040) compared with the rest condition	23.3
Mancı et al. [58]	*N* = 19 19 male 27.0 ± 4.2	A	Valorant Amateurs	Game performance (time to eliminate 50 targets) Go/No-Go test (reaction time, accuracy), Tracking test (cursor distance) Prefrontal-cortex hemodynamics via fNIRS	Maximal cycling sprint 4:30 min maximal load (30 s)	Quasi-experimental design with an age-matched control group	PE improved game performance in both groups. Amateur esports players demonstrated higher accuracy 30 min after exercise, while e-athletes responded faster (p = 0.033). In addition, both groups performed better on the Valorant task 30 min post-exercise compared with 5 min post-exercise	22.0
Rightmire et al. [59]	*N* = 28 26 male 22.0 ± 2.0	A	SSBU Amateurs	Game performance (win rate) Trail Making Test VO₂_max_, body composition, heart rate	HIIT on treadmill 30 min 90–95% of HRₘₐₓ (4*30 s)	Randomized controlled trial, parallel-group design	PE improved a participant’s likelihood of winning the second match if the first match (baseline) was lost (p = 0.006). The estimated log-odds value indicates that the probability of winning after acute exercise doubled from 6 to 12%	22.7
Zhang et al. [49]	*N* = 34 28 men 18.4 ± 0.8 y	A	Multi-game* Pros	Cognitive tasks from Human Benchmark (sequence memory, chimp test, aim trainer for speed/accuracy, visual memory, reaction time)	Moderate cycling 30 min 64–76% of HRₘₐₓ	Randomized controlled crossover trial	The PE groups showed significant improvements immediately after the intervention compared with baseline in speed–accuracy (p < 0.001, p = 0.005), visual memory (p < 0.001, p = 0.015), and reaction time (p < 0.001, p < 0.001). The training group also demonstrated significant improvements 30 min post-intervention compared with baseline in speed–accuracy (p = 0.002, p = 0.009) and reaction time (p = 0.009)	23.0
Wachholz et al. [61]	*N* = 28 28 male 27.5 ± 8.3	C	Multi-game* Amateurs	AimLab grid shot and track point, single reaction time, fourfold reaction time, anticipation task Overall coordination (BlazePods shuttle test), grip strength (dynamometer)	HIIT—whole-body and esports-specific 3 × 40 min per week over 8 weeks No details on intensity	Randomized controlled trial, parallel-group design	Both training groups showed significantly improved overall coordination compared with the control group (p = 0.035). Grip strength also improved significantly in the two training groups (p = 0.007). None of the other proposed variables related to esports performance demonstrated significant changes in response to the interventions	24.3
Lachowicz et al. [60]	*N* = 66 43 male 22.7 ± 0.66	C	Multi-game* Amateurs	Cognitrone (vienna test system, VTS) and Color Trail Test (CTT-1 and CTT-2)	Beat Saber (VR application with PE focus) 15min for 8 weekdays No details on intensity	Two-group, randomized, single-blind controlled trial (pilot)	The VR PE training significantly enhanced concentration performance and alternating attention vs. control (p < 0.001)	22.3
Lachowicz et al. [53]	*N* = 93 55 male 23.9 ± 3.6	C	Multi-game* Amateurs	Cognitrone (Vienna Test System, VTS) and Color Trail Test (CTT-1 and CTT-2	Beat Saber (VR application with PE focus) Group 1: 15min for 8 weekdays Group 2: 15min for 28 weekdays No details on intensity	Randomized controlled trial, parallel-group design	Both short- and long-term VR PE training improved concentration and executive functions (p < 0.001), with more correct responses, fewer errors, and faster task completion. Effects remained stable at follow-up, while controls showed no change. Notably, the short-term program was as effective as the longer one, suggesting no added benefit from extended training	23.7
Rightmire et al. [62]	*N* = 26 24 male 21.6 ± 1.9	A&C	SSBU Amateurs	Game performance (win proportion) Trail Making Tests A and B VO₂_max_, body composition, heart rate	HIIT training 3 × 30 min per week for 8 weeks 75–95% of HRₘₐₓ	Randomized controlled trial, parallel-group design	HIIT and acute bouts of PE improved reaction accuracy, reaction time, and fitness variables vs. control (p < 0.050). Retained short-term benefits post-training	23.3
Nicholson et al. [52]	*N* = 6 6 males 20.5 ± 3.0	C	LoL, overwatch 2 Pros	Corsi block-tapping task, color word stroop task, parametric go/no-go task Functional near-infrared spectroscopy in prefrontal-cortex ECG-based heart rate variability Graded treadmill test	HIIT treadmill training 3 × 30 min per week for 10 weeks 75–95% of HRₘₐₓ	Single-group pre–post design (no control group)	Weight and body mass index remained unchanged (p > 0.050). Executive functions improved, particularly sustained attention, response inhibition, and reaction time (p < 0.050), while working memory was maintained. Heart rate variables showed significant increases with large effect sizes	15.7

^*^ Multi-Game indicates whether multiple game titles were included in the study design or played by the participants. Abbreviations: *A* Acute, *C* Chronic, *y* years, *LoL* League of Legends, *SSBU* Super Smash Bros. Ultimate, *Pros* Professionals, *HR*_*max*_ maximum heart rate, *PE* Physical Exercise, *VR* Virtual Reality, *HIIT* High-Intensity Interval Training, *D&B Downs and Black*

### Effects of Acute Physical Exercise Training on Esports Performance

Eight studies investigated short-term effects. DiFrancisco-Donoghue et al. (2021) reported that a 6-min walk break significantly improved executive function compared to rest and continuous play, while rest breaks led to the poorest performance [54]. De las Heras et al. (2020) demonstrated that 15 min of HIIT cycling increased aiming accuracy by 75% and elimination rate by 9% in League of Legends [55]. The study by Zhang et al. (2023) found that 30 min of moderate cycling improved reaction time, visual memory, and speed–accuracy in professional players, with some effects lasting up to 30 min post-intervention [49]. Dos Santos et al. (2024) reported that moderate cycling reduced time to complete a CS:GO test by 7.6% while maintaining accuracy and improving precision [56]. Mancı et al. (2024) showed that a 30-s sprint enhanced both cognitive and gaming performance in Valorant [58]. Rightmire et al. (2024) found that HIIT training before competition doubled win probability in Super Smash Bros for previously unsuccessful players [59]. Another study by Rightmire et al. (2025) found an effect of acute PE on short-term improvements in esports-relevant performance when combined with chronic PE. Particularly in measures of attention and reaction speed, indicating immediate cognitive benefits following exercise [62]. One study by Gaige et al. (2024) reported mixed results: fatigue increased immediately after training, but improved goal-scoring rates later in the tournament [57]. Hence, improvements in executive function, gaming accuracy and precision, cognitive performance, and win probability, with one study noting initial fatigue but later better goal-scoring were observed.

### Effects of Chronic Physical Exercise on Esports Performance

**Fig. 2:**
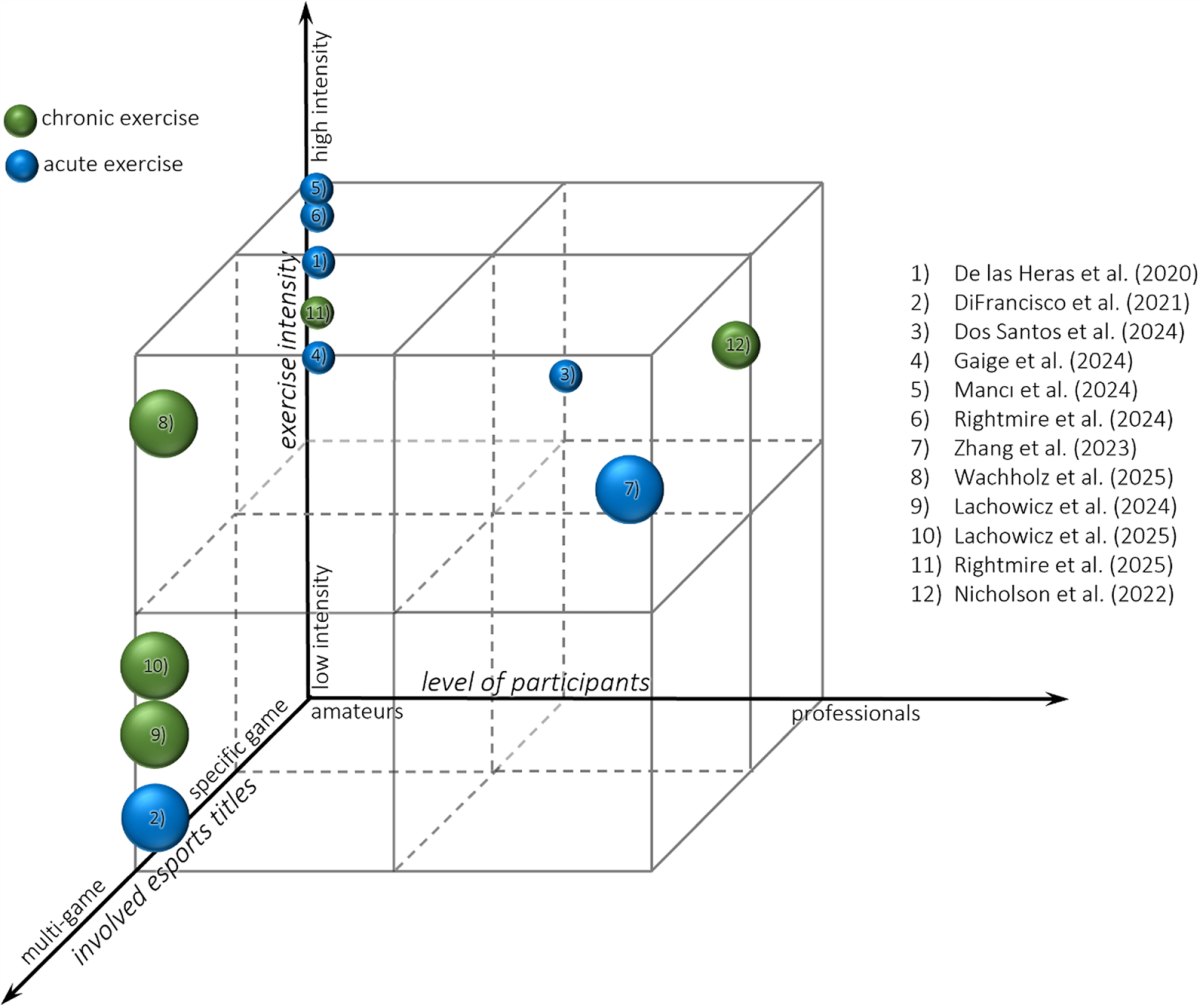
Three-dimensional visualization of included intervention studies examining the effects of PE on esports performance. The *x*-axis describes the level of evaluated participants (from amateur to professional players), the exercise intensity is shown on the *y*-axis (from low to high) and the z-axis represents the involved esports titles (from multi-game investigations to single-game designs).Each sphere denotes one study, with blue spheres indicating acute exercise interventions and green spheres representing chronic exercise interventions. The size of each sphere reflects the position along the “involved esports titles” axis, with smaller spheres representing studies on specific games and larger spheres representing studies covering multiple game titles. Numbers within the spheres refer to the studies listed on the right-hand side

Five studies evaluated longer-term interventions. Rightmire et al. (2025) conducted an 8-week intervention using different modalities serving as chronic PE training. No significant effect of chronic endurance training was observed. However, the endurance training effects on esports performance became evident only when chronic PE was complemented by an acute HIIT intervention [62]. Wachholz et al. (2025) applied an 8-week HIIT intervention (with and without esports-specific training) and found improvements in coordination and grip strength, but again no direct impact on esports performance [61]. Lachowicz et al. (2024) tested short-term VR-based training in amateur players, in which the experimental group completed eight 15-min Beat Saber sessions, which significantly improved concentration and alternating attention compared to controls, with effects sustained at follow-up [60]. This approach was extended by Lachowicz et al. (2025) by comparing short- and long-term VR training [53]. Both interventions enhanced concentration and executive functions, with more correct responses, fewer errors, and faster task completion. Effects remained stable at follow-up, while controls showed no change. Importantly, the short-term program was as effective as the longer one, suggesting no added benefit from extended training. Nicholson et al. (2024) conducted a pilot study with a 10-week HIIT program and found significant improvements in executive functions (e.g., inhibition, reaction time) and cardiovascular parameters, though no direct esports performance measures were included [52] (Fig. 2).

### Effects of PE Type, Duration, Intensity and Rest Periods

Five studies examining acute effects employed some form of cycling-based PE, either at moderate intensity [49, 56, 57] or as a HIIT variant [55, 58]. One additional acute study applied HIIT on a treadmill [59], while another used walking as the PE intervention [54]. Across all acute interventions, significant improvements in esports performance were observed. However, the assessment methods varied considerably—ranging from in-game performance indicators (e.g., number of eliminated targets [55, 56, 58], kill/death ratio [54], tournament outcomes [57, 59]) to executive function and other cognitive tasks [49]. In contrast, the studies investigating chronic PE interventions exclusively implemented HIIT-based training programs [52, 61, 62], or involved the VR application Beat Saber [53, 60], which promotes full-body engagement, particularly of the upper body. Reported outcomes included enhanced concentration [53, 60], executive functioning [52, 53], overall coordination, and grip strength [61]. Nevertheless, some studies found no significant effects of chronic PE on specific esports-related parameters, such as reaction time [61, 62].

The duration of acute PE interventions ranged from 6-min breaks [54] to 30-min sessions [49, 58, 59]. No consistent relationship between intervention duration and the magnitude of performance improvement was observed. In the chronic PE intervention studies, training sessions typically lasted 30 min, performed three times per week over a period of 8–10 weeks [52, 61, 62]. Studies employing the VR application Beat Saber as a PE intervention implemented 15-min sessions [53, 60], both of which reported improvements in concentration and executive functions. In contrast, the longer HIIT-based interventions produced mixed results, with some studies showing no significant effects on esports performance parameters [61, 62], while others reported notable improvements [52]. The intensity of the PE interventions was frequently reported across studies, ranging from “light”-intensity effort [54] to 75–95% of maximum capacity in the chronic interventions [52], and up to 90–95% of HR_max_ or maximal load in the acute studies [58, 59]. When comparing the included studies, no clear relationship emerged between PE intensity and outcomes on esports performance parameters. Moreover, four studies analyzed the timing between PE and esports performance. De las Heras et al. (2020) included a 20-min pause after HIIT, observing significant gains in accuracy and elimination rates [55]. Zhang et al. (2023) tested players immediately and 30 min after training, finding improvements in reaction time, speed–accuracy, and visual memory at both timepoints [49]. Mancı et al. (2024) compared effects at 5 and 30 min post-exercise, reporting increased accuracy after 30 min [58]. Gaige et al. (2024) included no pause and found initial fatigue, but improved scoring rates approximately 30 min later in tournament play [57].

## Discussion

This review sought to explore the effects that acute or chronic PE has on performance of e-athletes. The review found that both types of PE appear to have an improving effect on various esports performance indicators. Acute PE was investigated more frequently and revealed a consistently positive effect, as all included studies demonstrated significant improvements in esports-related cognitive or performance outcomes [49, 54–59]. In contrast, the findings from chronic PE interventions were more heterogeneous, with some studies reporting improvements in concentration [52, 53, 60], coordination [61], and executive functions [52], while others found no significant changes in reaction time or esports performance parameters [61, 62].

The type, duration, and intensity of PE appear to be less decisive for the observed benefits than previously [28] assumed. Instead, skill-specific interventions—such as VR-based exergames like Beat Saber—may provide superior benefits for two reasons: i) they engage task-relevant perceptual-motor skills (e.g., visuomotor coordination, processing speed, and reaction time), closely resembling the demands of esports gameplay [26], while simultaneously involving whole-body movement and ii) they may enhance training motivation and adherence due to their gamified and immersive nature [63, 64]. However, and although Lachowicz et al. [53, 60] demonstrated significant cognitive improvements following the VR-based Beat Saber intervention compared to passive controls, the design does not allow disentangling whether these effects were driven primarily by the PE component, the VR-mediated cognitive stimulation, or their interaction. Future studies including VR-only and PE-only comparison groups are, therefore, needed to isolate the underlying mechanisms.

Drawing on mechanistic insights from traditional exercise science, it can be argued that the observed effects (e.g., heart rate elevation, VO₂ increase, improved reaction times, neurophysiological activation) address the same biological and physiological mechanisms known to enhance performance in traditional sports and cognitive tasks [27, 28]. PE exerts its influence through multiple, well-documented physiological and psychological pathways. First, it leads to improved cerebral blood flow and oxygen delivery, as increased cardiac output ensures that more oxygen and nutrients reach the brain, resulting in higher efficiency in attention, reaction speed, and working memory [65–67]. Second, neurotransmitters play a critical role, as regular PE enhances the release of dopamine, serotonin, and noradrenaline, which are key modulators of motivation, attention, and vigilance [68, 69]. It also elevates the level of Brain-Derived Neurotrophic Factor (BDNF), which promotes synaptic formation, neuronal plasticity, and learning processes [70]. Third, hippocampal and prefrontal-cortex plasticity is stimulated through PE, inducing structural adaptations that support memory, executive functions, and inhibitory control [30]. Fourth, PE contributes to greater stress resilience by training the autonomic nervous system, improving cortisol regulation, and thereby stabilizing performance under competition-related stress while reducing mental fatigue [71]. Fifth, energy and glucose metabolism efficiency in the brain improves with PE, allowing for more effective responses to cognitive demands and prolonged concentration during play [72]. Finally, known psychological benefits such as better sleep quality [73] and enhanced attentional control and executive functioning [29] complement these effects. To interpret these heterogeneous yet promising performance effects, it is necessary to draw on mechanistic insights from traditional exercise science. PE is known to influence the core cognitive abilities required for esports through several well-documented physiological and psychological pathways.

An important consideration in interpreting the present findings is the genre-specific nature of esports performance. Different game genres impose distinct cognitive and motor demands, which may moderate the effectiveness of PE. Prior research has shown that action and FPS games primarily rely on rapid attentional allocation, visuospatial processing, and perceptual decision-making [22, 24]. In contrast, strategy-based genres such as RTS and MOBA games seem to place greater emphasis on working memory, planning, and cognitive flexibility [23]. These differences suggest that the transfer effects of PE may not be uniform across esports titles. However, based on the results, acute PE improved performance in, e.g., two games—Valorant [58] and CS:GO [56]—related to FPS genre, as well as, e.g., an MOBA game like League of Legends [55]. From a mechanistic perspective, acute bouts of PE have been associated with transient improvements in attention and processing speed [29], which may be particularly beneficial for performance in fast-paced action games, where rapid reactions and sustained attention are critical. Conversely, chronic exercise interventions have been linked to enhancements in executive functions, including cognitive flexibility and working memory [30], which may be more relevant for strategy-oriented games requiring complex decision-making and long-term planning. However, results for the effects of chronic PE interventions were more heterogeneous. In addition to cognitive demands, physical and motor requirements may also differ across genres. While classical esports are often characterized by fine motor control, certain genres involve higher demands on hand–eye coordination, precision, and motor speed, which may interact with exercise-induced improvements in neuromuscular function and fatigue resistance [26]. Furthermore, the temporal structure of gameplay (e.g., continuous high-intensity action vs. intermittent strategic phases) may influence how acute or chronic PE affects in-game performance, which should be considered in future research.

From a public health and physical activity perspective, the known mechanisms and the findings of the systematic review emphasize that integrating PE as a complement to sedentary esports and gaming is evidence-based not only for improving performance, but also for promoting health and well-being. This dual benefit positions PE as a meaningful and arguably necessary component of esports training programs, aligning esports more closely with the conceptual framework of sports medicine. It is particularly noteworthy that rest intervals of up to 30 min between PE and esports performance did not diminish the observed positive effects [49, 55, 57, 58]. This finding underscores the practical relevance of PE interventions in real-world competitive settings, as e-athletes could feasibly incorporate short bouts of PE as preparatory routines prior to tournaments. Importantly, such interventions do not need to occur immediately before gameplay but can be scheduled within a realistic and manageable time frame, making their implementation in professional training contexts both practical and effective. Consequently, the current evidence supports the view that structured PE should be systematically implemented in esports, as it targets core physiological and cognitive mechanisms known from traditional athletic training. This could also open new dimensions in the ongoing debate if esports should be considered as a sport [12, 13, 17, 17], as it provides empirical evidence that PE contributes to performance enhancement through mechanisms identical to those observed in physical sports [74]. At the same time, it must be noted that the term “esports performance” remains problematic within the scientific literature, as measurement approaches differ significantly between studies and no standardized assessment protocols currently exist. The wide variety of game genres and performance metrics complicates comparisons across studies and may contribute to the heterogeneity of findings. In addition, the Downs and Black checklist scores [47] indicate that the included studies are of moderate to high quality. However, further research with excellent methodological quality, rigorous study designs, and larger sample sizes is needed. In the context of esports research, future studies should aim to establish standardized performance metrics, and ensure improved validity to better capture the complex nature of esports performance. The lack of female participants and the scarcity of research focusing on women in esports represent another significant gap in the literature that should be addressed. Moreover, the lack of a universal terminology likely caused relevant studies to remain undetected during database screening [7].

Hence, future research should, therefore, aim to standardize esports performance parameters and extend the duration of chronic PE interventions. Moreover, the integration of neurophysiological and psychometric outcomes could confirm the hypothesized mechanisms linking PE, cognitive enhancement, and esports performance. Further studies should aim to experimentally evaluate the proposed physiological and neurocognitive mechanisms and provide empirical support for their hypothetical influence within the esports context. This includes investigating how changes in cerebral blood flow, neurotransmitter activity, and neuroplasticity translate into measurable improvements in esports-related cognitive and performance outcomes. Moreover, future research should work toward standardizing esports performance parameters, as the current heterogeneity in outcome measures limits comparability across studies. Establishing validated and genre-independent assessment protocols would enable stronger conclusions regarding the impact of PE on esports performance. Finally, researchers are encouraged to conduct long-term PE interventions to determine the persistence and practical relevance of these effects under realistic training conditions. Such efforts would help bridge the gap between exercise physiology and professional esports, advancing the evidence base for integrating structured physical training into esports practice.

### Limitations

Several limitations should be acknowledged when interpreting the findings of this review. First, the included studies investigated a wide range of esports genres and involved heterogeneous participant populations, ranging from amateur to professional players. This variability limits the direct comparability of results. However, such diversity also reflects the real-world complexity and ecological validity of the esports ecosystem, where different game genres impose distinct physical and cognitive demands. Moreover, many of the included studies differed substantially in how potential confounders were addressed, which complicates the interpretation and comparability of findings. Variables such as baseline physical fitness [30], prior gaming experience [75], sleep [76], fatigue [77], and habitual physical activity [34] were not consistently controlled for, despite their known influence on both cognitive performance and esports outcomes. In addition, the limited use of covariates and insufficient reporting of participant characteristics in several studies further restricted the ability to isolate the specific effects of PE. Heterogeneity in study designs—including variation in intervention protocols (type, intensity, and duration of exercise), outcome measures, and timing of assessments—further limits comparability across studies. The inconsistent inclusion of control groups and, in some cases, small sample sizes also reduce internal validity and increase the risk-of-biased estimates. These methodological limitations have important implications for the reliability and generalizability of the findings. While preliminary evidence suggests potential benefits of PE for esports performance and health, the strength of these conclusions is constrained by the underlying study quality and the extent to which confounding variables were adequately controlled. Hence, future research should prioritize more rigorous experimental designs, including randomized controlled trials with clearly defined control conditions. Standardized reporting of key covariates—such as physical fitness, gaming experience, and lifestyle factors—should be implemented to improve comparability across studies. In addition, the parameters used to assess esports performance differed considerably across studies, ranging from in-game performance metrics (e.g., kill/death ratio, aiming accuracy) to cognitive outcomes (e.g., executive function, reaction time). Although this diversity complicates quantitative comparison, it simultaneously highlights the multidimensional nature of esports performance, encompassing both cognitive and motor domains. Recognizing this heterogeneity is essential for guiding future efforts toward standardized assessment protocols that can capture esports performance in a more consistent and theoretically grounded way. Addressing these methodological considerations will be essential for advancing the evidence base and developing robust, evidence-informed training recommendations in esports. Second, many relevant studies were identified through hand-searching, as several esports-related journals are not yet indexed in major scientific databases, such as Web of Science or PubMed. While this could be seen as a limitation, it is important to note that the esports research field is relatively young, and the inclusion of these emerging outlets ensured a more comprehensive and up-to-date evidence base. Excluding such studies would have risked omitting relevant empirical work published in newly established journals. Third, the methodological quality of the included studies was mixed, as indicated by the Downs and Black [47] assessment. Yet, the average methodological quality was good, and many studies showed sound experimental designs and clear reporting of key variables. The inclusion of studies with varying quality levels also allowed for a more realistic overview of the current research landscape and helped identify methodological gaps that future studies can address.

## Conclusions

This review provides converging evidence that structured PE—both acute and chronic—can enhance esports performance through improvements in attention, executive function, visuomotor coordination, and actual game performance. While the exact modality, duration, and intensity of PE appear less decisive, interventions that combine an aerobic load with optionally integrated task-specific movement patterns tend to produce the most consistent and transferable benefits. Importantly, the persistence of positive effects even after rest intervals highlights the practical applicability of exercise as a preparatory routine before competition. From a sports medicine perspective, these findings extend established physiological and cognitive mechanisms of traditional athletic training, such as increased neuroplasticity, neurotransmitter balance, and autonomic regulation to the digital performance domain. Integrating PE into esports training, therefore, not only promotes performance optimization but also contributes to health maintenance in a population characterized by prolonged sedentary behavior and high mental strain. By establishing evidence-based exercise protocols for e-athletes, sports medicine can play a pivotal role in shaping sustainable and health-oriented models of esports performance development.

## Data Availability

All extracted data for the systematic review is included within this manuscript.

## Abbreviations

BDNF: Brain-Derived Neurotrophic Factor
CS:GO: Counter-Strike:Global Offensive
FPS: First-Person Shooter
HIIT: High-Intensity Interval Training
MOBA: Multiplayer Online Battle Arena
PE: Physical Exercise
PICOS: Population, Intervention, Comparator, Outcome, ans Study Design
PRISMA: Preferred Reporting Items for Systematic Reviews and Meta-Analysis
RCT: Randomized Controlled Trial
RTS: Real-Time Strategy
VR: Virtual Reality

## Declarations

### Declaration of AI Usage

The Large Language Model ChatGPT (OpenAI, San Francisco, CA, USA) was used to improve the readability and language of the manuscript. In accordance with the authorship policy of Sports Medicine— Open, the model does not meet the criteria for authorship, as accountability for the content cannot be attributed to artificial intelligence tools.
